# Pulmonary Venous Obstruction in Cancer Patients

**DOI:** 10.1155/2015/210916

**Published:** 2015-09-06

**Authors:** Chuang-Chi Liaw, Hung Chang, Tsai-Sheng Yang, Ming-Sheng Wen

**Affiliations:** ^1^Division of Hemato-Oncology, Department of Internal Medicine, Chang-Gung Memorial Hospital and Chang-Gung University College of Medicine, Taoyuan 33305, Taiwan; ^2^Division of Cardiology, Department of Internal Medicine, Chang-Gung Memorial Hospital and Chang-Gung University College of Medicine, Taoyuan 33305, Taiwan

## Abstract

*Background*. We study the clinical significance and management of pulmonary venous obstruction in cancer patients. *Methods*. We conducted a prospective cohort study to characterize the syndrome that we term “pulmonary vein obstruction syndrome” (PVOS) between January 2005 and March 2014. The criteria for inclusion were (1) episodes of shortness of breath; (2) chest X-ray showing abnormal pulmonary hilum shadow with or without presence of pulmonary edema and/or pleural effusion; (3) CT scan demonstrating pulmonary vein thrombosis/tumor with or without tumor around the vein. *Results*. Two hundred and twenty-two patients developed PVOS. Shortness of breath was the main symptom, which was aggravated by chemotherapy in 28 (13%), and medical/surgical procedures in 21 (9%) and showed diurnal change in intensity in 32 (14%). Chest X-rays all revealed abnormal pulmonary hilum shadows and presence of pulmonary edema in 194 (87%) and pleural effusion in 192 (86%). CT scans all showed pulmonary vein thrombosis/tumor (100%) and surrounding the pulmonary veins by tumor lesions in 140 patients (63%). PVOS was treated with low molecular weight heparin in combination with dexamethasone, and 66% of patients got clinical/image improvement. *Conclusion*. Physicians should be alert to PVOS when shortness of breath occurs and chest X-ray reveals abnormal pulmonary hilum shadows.

## 1. Introduction

Cancer cells can pass through a lung capillary and/or direct extension into pulmonary vein [1]. Tumor that extends into pulmonary veins may cause the pulmonary vein flow stasis, and/or vascular injury results in thrombosis generation [2–5]. Pulmonary veins infiltration by tumors or compression by affected lymph nodes result in venous stasis is also a potential reason to develop thrombosis [2–5]. Multiple pulmonary venous thrombosis/tumor is a potentially fatal condition. Impedance of blood flow from the pulmonary vein to the left atrium may cause pulmonary edema or pleural effusion.

In the present prospective case series study, we investigate the clinical significance of pulmonary vein obstruction in cancer patients and better characterize the syndrome that we term “pulmonary vein obstruction syndrome” (PVOS). The management of PVOS is also studied.

## 2. Materials and Methods

### 2.1. Patients

Between January 2005 and March 2014, we conduct a prospective case series study. Data collected from 1117 patients hospitalized in oncology wards of the Chang-Gung Memorial Hospital. Our data source mainly came from a single physician. The urological cancer was our area of expertise; most of these patients had urothelial carcinomas.

### 2.2. Diagnostic Criteria of PVOS

The criteria for PVOS diagnosis inclusion/diagnosis are listed as symptoms, chest X-ray findings, and CT findings, and that all 3 were required. The criteria for inclusion were (1) episodes of shortness of breath; (2) chest X-ray showing unilateral or bilateral abnormal pulmonary hilum shadow with or without presence of pulmonary edema and/or pleural effusion; (3) CT (computed tomography) scan demonstrating pulmonary vein thrombosis/tumor with or without lesions sticking to the outer vein surface. When dyspnea occurred and chest X-ray shows abnormal hilum shadow, CT scan was traced to detect pulmonary vein thrombosis/tumor. The majority of patients did a CT scan before the onset of symptom. But CT scans were not sensitive enough to separate thrombosis from tumor embolism. No patients had prior congestive heart failure history. The study was approved by the hospital ethics committee.

### 2.3. Clinical Investigation

The characteristics of PVOS included the presence of acute respiratory distress, combined with other thromboembolic complications and with other paraneoplastic syndromes. Acute respiratory distress was described as aggravated by chemotherapy, aggravated by medical/surgical procedures, and subject to diurnal fluctuation in intensity. Common thromboembolism-associated complications included consciousness loss/mental change [6, 7], paraneoplastic pain, and iliofemoral venous thrombosis symptoms. Paraneoplastic pain was defined as breakthrough pain occurring in the absence of an identifiable precipitating cause [8]. Cerebral thromboembolic complication and/or paraneoplastic pain in most patients were clinically suspected because of difficulty in definite diagnosis. Paraneoplastic syndromes included neoplastic fever (tumor-related fever with good response to naproxen test) [9], cachexia syndrome (simultaneous presence of weight loss > 5% within 6 months, reduced food intake, and muscle wasting) [10].

### 2.4. Laboratory Study

The D-dimer test, complete blood counts, liver function test, renal function test as checked in all patients with PVOS, APTT (active partial thromboplastin time) and PT (prothrombin time), calcium, C-reactive protein, blood gas, and pleural effusion study were checked in selected patients when diagnosing PVOS. The cutoff D-dimer value was 500 ng/mL. Paraneoplastic syndromes included hypercalcemia (serum calcium level more than 11 mg/dL), leukemoid reaction (peripheral count to more than 20,000/*μ*L without evidence of infection or leukemia), and prerenal azotemia was defined as BUN-to-creatinine ratio greater than 20.

### 2.5. Image Study

Chest plain film findings included the location of abnormal pulmonary hilum shadows, presence of pulmonary edema, and presence of pleural effusion. CT scan findings included pulmonary vein obstruction sites, presence of pulmonary embolism, and presence of pleural effusion. Echocardiography and lung ventilation/perfusion scan were performed in selected patients when diagnosing PVOS.

### 2.6. Therapy

Treatment included subcutaneous injection of low molecular weight heparin (LMWH) either Fraxiparin (GlaxoSmithKline) or Enoxaparin (Sanofi-Aventis), intravenous dexamethasone, and intravenous fluids with or without furosemide when PVOS with acute respiratory distress occurred. Further use of chemotherapy or targeted therapy or hormone therapy depended on the patient's condition. CT scans were obtained from the hospital picture archiving and communication system (PACS).

### 2.7. Statistical Methods

Continuous data (presented as mean ± standard deviation) were used for D-dimer, C-reactive protein, BUN, creatinine, and BUN-to-creatinine ratio analysis. Survival was calculated from the time of the diagnosis of PVOS to death. Survival curves were determined using Kaplan-Meier methods. The significance of difference between survival curves was measured by log-rank test.

## 3. Results

### 3.1. Patient Characteristics

Of 1117 patients, 222 patients (20%) were documented to have PVOS. The data for 222 consecutive cancer patients (139 men and 83 women; 27–93 years old; median age, 69) was collected for evaluation of PVOS. The patients' characteristics and important laboratory and imaging findings of PVOS were shown in Table 1. PVOS occurred in patients with various metastatic tumors. One hundred and sixty-seven patients (75%) had an Eastern Cooperative Oncology Group (ECOG) performance status of 2 or greater. Common association with thromboembolic complications occurred in 146 patients (66%): consciousness disturbance (*n* = 103), paraneoplastic pain (*n* = 53), and iliofemoral venous thrombosis symptom (*n* = 16). Of them, 62 associated with multiple thromboembolic presentations. Of 103 patients with consciousness disturbance, 16 had CT scan- or magnetic resonance imaging- (MRI-) evidence of cerebral infarction and/or their angiographic proven. Common association with paraneoplastic syndromes occurred in 101 patients (45%): cachexia syndrome (*n* = 79), neoplastic fever (*n* = 23), leukemic-like reactions (*n* = 18), hypercalcemia (*n* = 4), and lactic acidosis (*n* = 3). Of them, 15 had multiple syndromes.

### 3.2. Clinical Outcome

Shortness of breath was the main symptom. Acute respiratory distress (*n* = 222) was aggravated by chemotherapy (*n* = 28; 13%) and medical/surgical procedures (*n* = 21; 9%) and fluctuated diurnally in intensity (*n* = 32, 14%). Blood gas tests in 126 patients found acidity (pH less than 7.3) in 24 (19%), PaCO_2_ > 50 mmHg in 23 (18%), PaO_2_ < 60 mmHg in 36 (29%), HCO_3_− < 18 mEq/L in 35 (28%), and SaO_2_ < 90% in 35 (28%). Pleural effusion tests in 32 patients found exudate in 28 (88%), erythrocyte count < 500 in 14 (44%), leukocyte count < 500 in 22 (69%), and lymphocytes predominant in 25 (78%).

D-dimer and complete blood counts were checked in all patients. Mean D-dimer value was 3354 ± 2187 ng/mL (265 to greater than 10,000 ng/mL). D-dimer values 1001–3000 ng/mL in 35% of patients was the most common. There were 132 patients (59%) with hemoglobin levels below 10 g/dL, 95 patients (43%) with elevated white blood counts (>10,000/*μ*L), and 21 patients (9%) with decreased platelet counts (<100,000/*μ*L). APTT (active partial thromboplastin time) was checked in 100 patients and PT (prothrombin time) was checked in 109 patients; of these, 34 (34%) had APTT values above 36 seconds and 29 (27%) had PT values above 15 seconds. Albumin values were below 3.0 g/dL in 78 (41%) of the 189 patients. C-reactive protein was monitored in 111 patients. Mean C-reactive protein value was 114 ± 96 mg/L (0.7 to 384 mg/L). Of these 105 patients (95%) with elevation. Renal insufficiency was detected in 30 patients (16%). Of them mean BUN value, creatinine value, and BUN-to-creatinine ratio were 79.3 ± 41.6 mg/dL (36.3 to 163 mg/dL), 3.7 ± 2.7 mg/dL (0.65 to 12.3 mg/dL), and 21.4 ± 15.5 (6.9 to 60.5), respectively.

**Figure 1:**
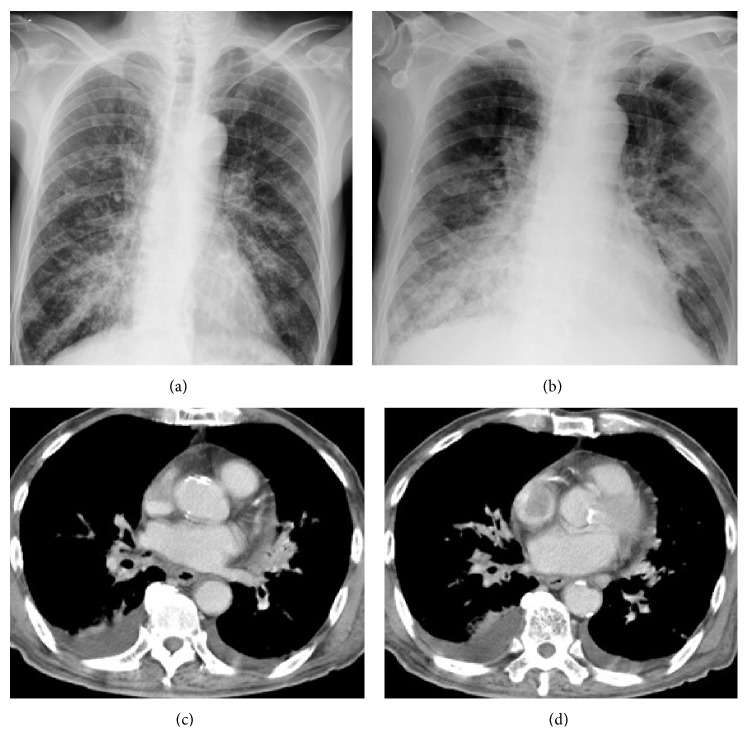
Pulmonary vein obstructive syndrome (PVOS). A 78-year-old man with rectal cancer. Chest X-ray (a) before and (b) at the onset of PVOS showed right low lung and pulmonary hilum increase haziness. CT scan ((c) and (d)) revealed tumor/thrombosis located in the bilateral superior and inferior pulmonary veins.

**Figure 2:**
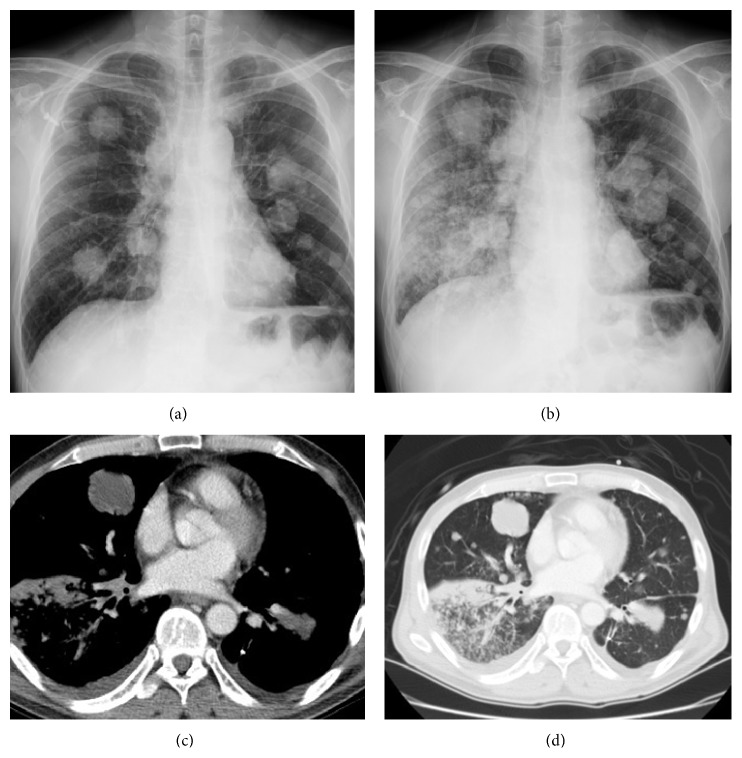
Pulmonary vein obstructive syndrome (PVOS). A 54-year-old man with bladder cancer. Chest X-ray (a) before and (b) at the onset of PVOS showed right low lung and hilum increase haziness. CT scan ((c) and (d)) revealed tumor/thrombosis located in right inferior pulmonary veins with peripheral extension.

**Figure 3:**
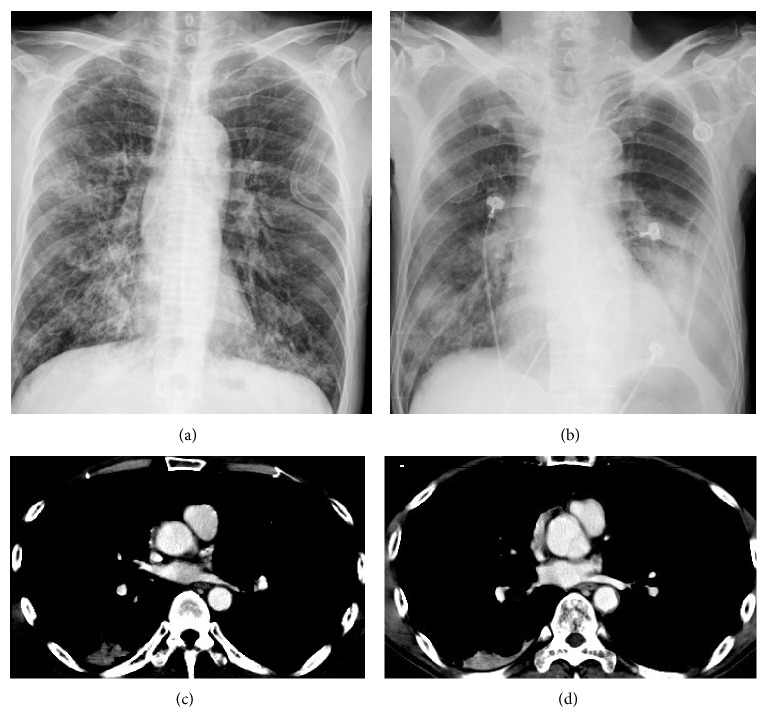
Pulmonary vein obstructive syndrome (PVOS). A 51-year-old man with oral cancer. Chest X-ray (a) before and (b) at the onset of PVOS showed a left low lung and hilum increase haziness. CT scan ((c) and (d)) revealed tumor/thrombosis located in superior and inferior pulmonary veins with left atrium extension.

**Figure 4:**
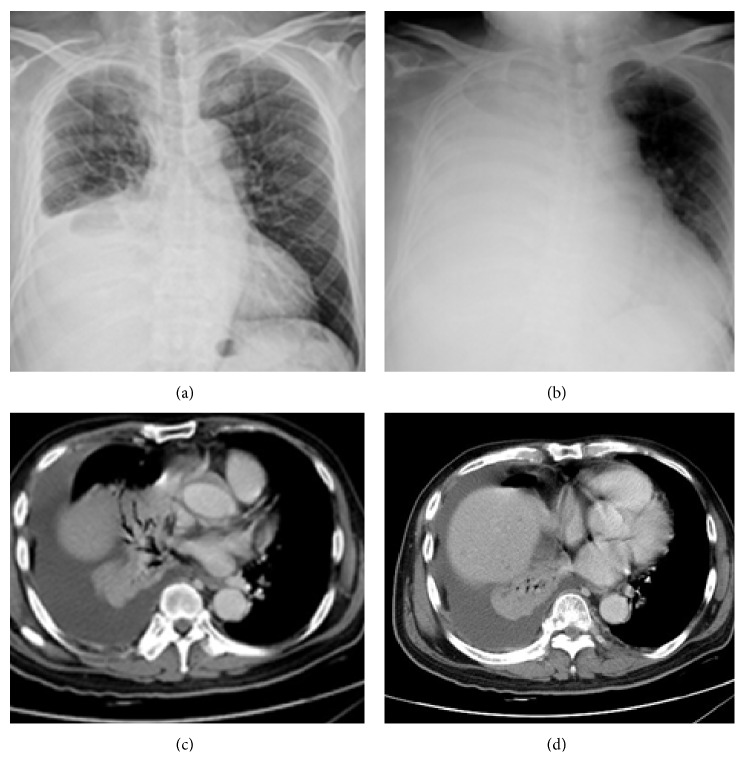
Pulmonary vein obstructive syndrome (PVOS). A 61-year-old man with lung cancer. Chest X-ray (a) before and (b) at the onset of PVOS showed right low lung increase haziness to total opacity. CT scan ((c) and (d)) revealed lung tumor and atelectatic lesions surrounding the superior and inferior pulmonary veins with right pleural effusion.

Chest plain X-rays of 222 patients before and at the onset of PVOS were shown in Figures 1(a), 1(b), 2(a), 2(b), 3(a), 3(b), 4(a), and 4(b). All revealed an increase pulmonary hilum shadows when PVOS developed. Abnormal hilum shadows were bilateral in 175 (79%) and unilateral in 47 (21%) and present in both lobes in 186 (84%), upper lobes in 19 (9%), and lower lobes in 17 (8%). Chest plain X-rays showed pulmonary edema in 194 patients (87%) and pleural effusion in 192 patients (86%) (Figures 1(b), 2(b), 3(b), and 4(b)).

CT scans that revealed tumor/thrombosis located in pulmonary veins were shown in Figures 1(c), 1(d), 2(c), 2(d), 3(c), 3(d), 4(c), and 4(d). The separate time between doing CT scan and detecting abnormal hilum shadows by chest films due to PVOS was 149 patients (67%) within 1 month, 46 (21%) in 1-2 months, 20 (9%) in 2-3 months, and 7 (3%) more than 3 months. All demonstrated pulmonary vein thrombosis or tumor. Tumor or atelectatic lesions surrounding the pulmonary vein (Figures 4(c) and 4(d)) were seen in 140 (63%). Pulmonary vein obstructions were present bilaterally in 204 (92%) and unilaterally in 18 (8%), in both the superior and inferior pulmonary vein in 203 (91%), in the superior pulmonary vein only in 11 (5%), and inferior pulmonary vein only in 8 (4%). Chest CT scan detected pleural effusion in 155 patients (70%), pulmonary artery embolism in 70 patients (32%), peripheral tumor/thrombi lesions (Figures 2(c) and 2(d)) in 72 patients (32%), and cardiac tumor/thrombi lesions (Figures 3(c) and 3(d)) in 7 patients (3%), and lung ventilation-perfusion scan in 5 patients found two (40%) with a pulmonary embolism and echocardiography in 29 patients found 14 (48%) with left atrium enlargement (≥38 mm).

### 3.3. Treatment Outcome and Survival Data

LMWH therapy was given to 170 patients with PVOS, including 113 on Fraxiparin (3800 IU or 5700 IU daily) and 57 on Enoxaparin (6000 IU daily). And intravenous dexamethasone was also used in 133 patients. Symptoms and/or image improvement in 113 (66%) including 99 of those treated with dexamethasone. Of them, 37 patients continued their LMWH for secondary prevention.

Of 69 patients who received therapy, including chemotherapy in 59, targeted therapy in 9, and hormone therapy in one, 46 (68%) had disease control. PVOS developed again after disease progression in 32 patients (74%), including 20 with their LMWH for secondary prevention.

**Figure 5:**
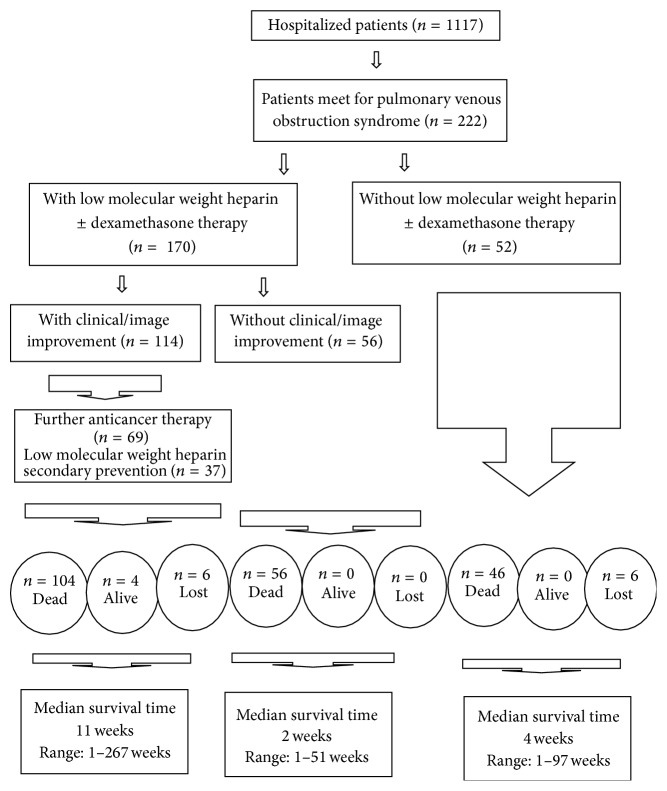
Flowchart of 222 cancer patients with pulmonary vein obstruction syndrome (PVOS).

Follow-up periods ranged from 1 day to 267 weeks. Besides the fact that 12 patients were lost to follow-up, 210 patients could be followed until death or up to the present. Four patients were still alive. Median overall survival time by Kaplan-Meier methods was 5 weeks. Three-month, 6-month, 1-year, and 2-year-survival probabilities were 30%, 13%, 9%, and 1%, respectively. For 52 patients not receiving LMWH therapy patients, median overall survival time was 4 weeks. For 170 patients receiving LMWH ± dexamethasone therapy showed clinical/image improvement in 114 patients. The composite survival rate for those with clinical/image improvement was superior to those without clinical/image improvement. The median survival rate was 11 weeks versus 2 weeks by log-rank test (*P* = 0.001). Flow chart of 222 patients with PVOS was shown in Figure 5.

The predeath status could be identified in 198 patients as respiratory failure (*n* = 90; 45%) and consciousness loss (*n* = 108; 55%). Sixty-three patients (32%) died of septicemia and/or febrile neutropenia, including 34 with respiratory failure and 28 with consciousness loss.

## 4. Discussion

PVOS occurred in 20% of our hospitalized patients with various malignancies.

PVOS is frequently associated with other thromboembolic complications and is a Trousseau's syndrome. Trouseau's syndromein is characterixed by spontaneous, multiple, recurrent, and migratory venous thrombosis, and arterial emboli [11–13]. A few patients also exhibit other paraneoplastic syndromes related to cytokine production [8–10].

Virchow describes the three elements, including venous stasis, endothelial injury, and hypercoagulability that are thought to contribute to venous thromboembolism (VTE) [14, 15]. The mechanism of prothrombotic state is particularly complex in cancer patients. Cancer cells can activate the hemostatic system through the expression of adhesion molecules, release of inflammatory cytokines, and production of hemostatic factors [16–20]. Activation of blood coagulation results in thrombin generation and intravascular fibrin formation [16–20]. Thrombin-activated tumor cell adhesion to host cells also enhances tumor cell growth, tumor cell seeding, and spontaneous metastasis and stimulates tumor angiogenesis [16–19]. Once cancer cells enter into and/or approach to the pulmonary vein, they result in blood flow stasis and vascular injury. Cancer patients can be in a hypercoagulable status. But the cytokine production is the primary culprit to the development of PVOS. The increase cytokine production among cancer patients have been well recognized in sepsis, surgery/medical procedures and chemotherapy [20–22].

Three lines of evidence are presented in the study to indicate that PVOS is a real model of the thromboembolic complication [16, 17]. First, CT scans demonstrated pulmonary vein thrombosis/tumor. Tumor surrounding the pulmonary vein was noted in 63% of patients. Tumor cells result in pulmonary vein injury and/or stasis is essential. Second, acute respiratory distress is aggravated by chemotherapy and medical/surgical procedures and fluctuates diurnally in intensity. Cancer itself, its treatments, and its complications can activate cytokine signaling pathways including those of nuclear factor kappa B (NF*κ*B) and p38 mitogen-activated protein kinase (MAPK) [23]. Circadian rhythms of cytokine release have been demonstrated in people with advanced neoplasms [24]. Third, D-dimer and CRP levels were elevated in 89% and 95%, respectively, of our patients, as occurs in cases of VTE and PE [25, 26]. Elevated D-dimer or CRP has been associated with increased risk of VTE [27].

A rise in hydrostatic pressure occurs due to blood flow through the pulmonary vein to the left atrium stasis. Symptoms such as pulmonary edema and pleural effusion develop. A few patients already had an oncological emergency (i.e., respiratory failure with hypoxemia, hypercapnia, decreased pH, or low SaO_2_). A complex cause of pleural effusion noted in PVOS patients, and 69% had leukocyte count less than 500 on the pleural fluids. The reason in part for the low leukocyte count is that, in PVOS (like heart failure), hydrostatic pressure is also increased.

Cancer can induce pulmonary embolism (PE) formation and thrombotic formation and can invade large veins [28–30]. Extensive tumor-associated thromboemboli in the pulmonary microvasculature have been reported in a cancer patient with dyspnea [28–30]. However, microscopic tumor embolism is rarely recognized before death. The diagnosis was often identified from postmortem examination [28–30]. PVOS can combined with pulmonary artery embolism; tumor/thrombi from pulmonary veins can extend peripherally [28, 29] or enter left atrium [31]. Pulmonary artery embolism, peripheral pulmonary tumor/thrombi lesion, and cardiac tumor/thrombi lesions were 32%, 32%, and 3% of our patients, respectively.

The appearance of abnormal pulmonary hilum shadows on chest plain X-ray films (which are due to pulmonary hypertension) is essential for raising suspicion of PVOS [32]. Pulmonary edema and/or pleural effusion on chest plain films indicate severe blockade of pulmonary veins. CT scans are vital for the diagnosis of PVOS and can show pulmonary vein thrombosis, tumor, and pulmonary vein stricture especially from their abnormal pulmonary hilum shadows [33]. Pulmonary edema and/or pleural effusion may be unilateral or bilateral and may arise from the superior pulmonary vein, inferior pulmonary vein, or both. Bilateral lung and pulmonary veins involvement are seen in most of our patients. In the present study, pleural effusion was detected in 86% of our cases on plain chest X-ray films but in only 70% on CT scans. The discrepancy may be due to the timing of the CT scan, which was usually performed before the PVOS attack. Echocardiography found left atrium enlargement (thought to be due to stenosis of the pulmonary vein distal to the left atrium) in approximately 48% of our cases. In cancer patients, it is important to recognize that early appearance on chest X-ray of abnormal pulmonary shadows can result in a misdiagnosis of lung infection and acute respiratory distress syndrome.

The principle of treatment is based on procoagulant mechanisms. LMWH is used to improve the hemostatic condition [34]. Cytokine and NF*κ*B inhibitors, such as dexamethasone, are used to suppress cytokine formation in acute stage [35]. Stress related to cytokines can influence the course of neoplastic diseases [36]. Preventing unnecessary procedures and calming the patients can reduce cytokine overproduction. Knowing aggravated factors can cause PVOS. When an infection occurs, antibiotics is given immediately. Adequate fluids supplement is necessary for maintaining good perfusion of vital organ (brain, coronary artery, and kidney) [6, 7, 37, 38]. The importance of cancer-associated hyper-coagulation is an etiology of acute ischemic stroke [6, 7] and probably a leading death in our patients. Renal insufficiency had been linked to increased mortality from the literature [38] and prerenal azotemia pattern in our 16% patients. Furosemide for preload reduction can provide immediate symptom relief when acute respiratory distress with pulmonary edema occurs. Blood gas analysis can help the assessment of disease condition. Maintenance of LMWH is suggested for secondary prevention [39, 40] and probably a survival benefit [41–43]. Underlying disease therapy can be treated if feasible. Clinical/image improved in 66% of our patients after LMWH with or without combination with dexamethasone. Disease control was achieved in 68% of these patients after further anticancer therapy. The survival rate for those with clinical/image improvement was superior to those without clinical/image improvement.

The outcome of PVOS was dismal (median survival time, 6 weeks). The cause of death was either respiratory failure or consciousness disturbance and related to thromboembolic complications. Two-thirds patients died of septicemia and/or febrile neutropenia. In the pathogenesis of sepsis, inflammation and coagulation play a pivotal literature [19]. Sepsis increases cytokine production and is considered to be an aggravating factor of thromboembolic complications.

Our study has several important limitations. First, the data was collected from prospective case cohort study in a single center mainly from a single physician. Secondary, image study included chest plain film and CT scan was probably not done simultaneously. Third, pulmonary vein thrombosis, tumor, or mixed type was difficult judge from CT scan. Fourth, consciousness/mental change related to thromboembolic complication seldom proven by image study. Fifth, the absence of tissue confirmation was either by biopsy or autopsy. Sixth, no cytokine study was demonstrated.

## 5. Conclusion

PVOS is a common but neglected thromboembolic complication. Physicians should be alert to PVOS when shortness of breath occurs and chest X-ray reveals abnormal pulmonary hilum shadows. Medical/surgical procedures, therapy, and infection can aggravate PVOS. Symptoms can be relieved by the administration of LMWH, dexamethasone, prevent unnecessary procedures, calm the patients, adequate fluids, furosemide given when there is acute respiratory distress, and antibiotics given immediately when there is an infection.

## Figures and Tables

**Table 1:** Characteristics and important laboratory and imaging findings of 222 cancer patients with pulmonary vein obstruction syndrome (PVOS).

Characteristics	Number of patients (%)
Age (years)	
Median (range)	69 (27–93)
Sex	
Male/female	139/83
Primary sites: number/total hospitalized number (%)	
All patients	222/1117 (20)
Urinary tract	80/395 (20)
Lung	31/115 (27)
Colorectum	15/67 (22)
Breast	18/58 (31)
Pancreas	8/46 (17)
Stomach	7/38 (18)
Prostate	7/29 (24)
Others	56/369 (13)
Performance status: number/total number (%)	
0-1	55/222 (25)
≥2	167/222 (75)
Associated with other thromboembolic complications: number/total number (%)	
Yes	146/222 (66)
No	76/222 (34)
Associated with other paraneoplastic syndromes: *number/total n*umber (*%*)	
Yes	101/222 (45)
No	121/222 (55)
Acute respiratory distress: number/total number	
Aggravated by chemotherapy	28/222 (13)
Aggravated by medical/surgical procedure	21/222 (9)
Showed diurnal rhythm	32/222 (14)
D-dimer (ng/mL): number/total number (%)	
≦1000	25/222 (11)
1001–3000	78/222 (35)
3001–5000	51/222 (23)
>5000	68/222 (31)
C-reactive protein (mg/L): number/total number	
≦10	6/111 (5)
>11	105/111 (95)
Chest plain film: number/total number	
Abnormal hilum shadow	222/222 (100)
By side	
Bilateral lung	175/222 (79)
Unilateral lung	47/222 (21)
By location	
Upper lung + lower lung	186/222 (84)
Upper lung only	19/222 (9)
Lower lung only	17/222 (8)
Pulmonary edema	194/222 (87)
Pleural effusion	192/222 (86)
CT scan: number/total number	
Pulmonary veins thrombosis/tumor	222/222 (100)
Surrounding pulmonary veins by tumor/atelectasis/consolidation	140/222 (63)
By side	
Bilateral lung	204/222 (92)
Unilateral lung	18/222 (8)
By location	
Both superior and inferior pulmonary vein	203/222 (91)
Superior pulmonary vein only	11/222 (5)
Inferior pulmonary vein only	8/222 (4)
Pulmonary artery emboli	70/222 (32)
Pleural effusion	155/222 (70)
